# The Acute Phase Response Is a Prominent Renal Proteome Change in Sepsis in Mice

**DOI:** 10.3390/ijms21010200

**Published:** 2019-12-27

**Authors:** Beáta Róka, Pál Tod, Tamás Kaucsár, Matej Vizovišek, Robert Vidmar, Boris Turk, Marko Fonović, Gábor Szénási, Péter Hamar

**Affiliations:** 1 Institute of Translational Medicine, Semmelweis University, 1094 Budapest, Hungary; beata.roka@gmail.com (B.R.); todpal90@gmail.com (P.T.); kaucsar.tamas@med.semmelweis-univ.hu (T.K.); szenasi.gabor@med.semmelweis-univ.hu (G.S.); 2 Institute for Translational Medicine, Medical School, University of Pécs, 7624 Pécs, Hungary; 3 Department of Biochemistry and Molecular and Structural Biology, Jožef Stefan Institute, 1000 Ljubljana, Slovenia; vizovisek@imsb.biol.ethz.ch (M.V.); Robert.Vidmar@ijs.si (R.V.); boris.turk@ijs.si (B.T.); marko.fonovic@ijs.si (M.F.); 4 Centre of Excellence for Integrated Approaches in Chemistry and Biology of Proteins, 1000 Ljubljana, Slovenia

**Keywords:** acute kidney injury (AKI), lipopolysaccharide (LPS), renal acute phase reaction (APR), acute phase proteins (APP), mouse

## Abstract

(1) Background: Sepsis-induced acute kidney injury (AKI) is the most common form of acute kidney injury (AKI). We studied the temporal profile of the sepsis-induced renal proteome changes. (2) Methods: Male mice were injected intraperitoneally with bacterial lipopolysaccharide (LPS) or saline (control). Renal proteome was studied by LC-MS/MS (ProteomeXchange: PXD014664) at the early phase (EP, 1.5 and 6 h after 40 mg/kg LPS) and the late phase (LP, 24 and 48 h after 10 mg/kg LPS) of LPS-induced AKI. Renal mRNA expression of acute phase proteins (APP) was assessed by qPCR. (3) Results: Renal proteome change was milder in EP vs. LP. APPs dominated the proteome in LP (proteins upregulated at least 4-fold (APPs/all): EP, 1.5 h: 0/10, 6 h: 1/10; LP, 24 h: 22/47, 48 h: 17/44). Lipocalin-2, complement C3, fibrinogen, haptoglobin and hemopexin were the most upregulated APPs. Renal mRNA expression preceded the APP changes with peak effects at 24 h, and indicated renal production of the majority of APPs. (4) Conclusions: Gene expression analysis revealed local production of APPs that commenced a few hours post injection and peaked at 24 h. This is the first demonstration of a massive, complex and coordinated acute phase response of the kidney involving several proteins not identified previously.

## 1. Introduction

Sepsis is estimated to be one of the leading causes of critical illness and mortality with constantly increasing incidence in intensive care units [1,2]. Sepsis is defined as an organ dysfunction of variable severities induced by a dysregulated immune response to an infection [3,4]. One of the organs determining early and late outcomes of sepsis is the kidney. Septic acute kidney injury (AKI) can be established within hours to days in up to 50% of intensive care unit patients [5]. In surviving patients, even mild or short episodes of AKI can predispose to an increased risk for developing chronic kidney disease and end-stage renal failure [4].

The acute phase response/reaction (APR) is part of the immune response to infections and tissue damage. Proteins whose plasma concentration is changed by at least 25% in response to pro-inflammatory stimuli are termed acute phase proteins (APP) [6,7,8]. They have a role in restoring homeostasis after inflammation [7]. Although APPs are thought to be primarily produced by the liver and secreted into the blood, they are also synthesized in other organs. Thus, APPs can contribute to local defense responses and repair mechanisms [8].

Plasma concentrations of APPs (e.g., C-reactive protein) are well-established markers for diagnosing and monitoring sepsis [9]. Lipocalin-2 (Lcn-2) also termed as neutrophil gelatinase-associated lipocalin (NGAL) is a sensitive marker of renal tubular injury [10,11,12,13]. However, none of these biomarkers is specific for sepsis or AKI [9,14]. For example, elevated serum Lcn-2 concentrations were observed in other pathologic conditions, such as cardiovascular [15] and gastrointestinal [16,17] diseases. Several other AKI biomarker candidates have been identified [14,18] but so far none of them was able to differentiate the various types of AKI with satisfactory specificity and sensitivity [14,19]. For this reason, there is an intensive effort to identify biomarkers or biomarker combinations that could be used for the early detection or follow-up of AKI [14].

The inflammatory and circulatory effects of sepsis are often modelled in rodents by administration of bacterial wall endotoxin (lipopolysaccharide: LPS) [20,21]. Increased renal production of some APPs, such as fibrinogen [22], ceruloplasmin [23], complement C3 [24,25], haptoglobin [26,27,28], hemopexin [29], serum amyloid A [23,27], beta-2-microglobulin [22], α-1-acid glycoprotein [23], and plasminogen activator inhibitor-1 [30] have been described in this model, while renal gene expression of serum albumin (a negative APP) was shown to be reduced [31]. Although these scarce and isolated findings demonstrate the renal production of some APPs in sepsis, a coordinated, complex APR of the kidney has not been evaluated before.

In the present study, we performed proteomic analysis to find relevant groups of proteins activated by LPS at early (1.5 h, 6 h) and late (24 h, and 48 h) time points after endotoxin administration in the kidney. Surprisingly, we found abundant representation of APPs among the most regulated proteins. Furthermore, qPCR measurements revealed that 14 out of these APPs were produced locally, in the injured kidney.

## 2. Results

### 2.1. LPS-Induced Severe Inflammation in the Kidney

All animals survived in the EP groups (40 mg/kg LPS), but one mouse died both in the LP24 and LP48 groups (10 mg/kg LPS) during the experiment.

**Figure 1:**
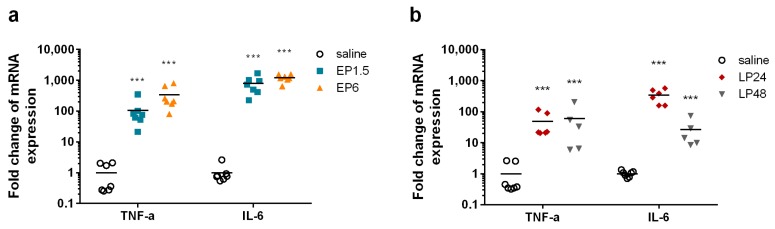
Fold changes of TNF-α and IL-6 mRNA relative to the saline-treated control kidneys in mice after lipopolysaccharide (LPS) administration. ***: *p* ≤ 0.001. (**a**) early phase (EP)1.5 and EP6 groups. (**b**) LP24 and LP48 groups.

Endotoxin significantly upregulated renal TNF-α and IL-6 mRNA production at all time points (Figure 1). Both LPS doses caused severe, maximal inflammatory response in the kidneys. IL-6 mRNA but not TNF-α mRNA started to decrease at 48 h.

### 2.2. LPS-Induced Tubular Damage in the Kidney

**Figure 2:**
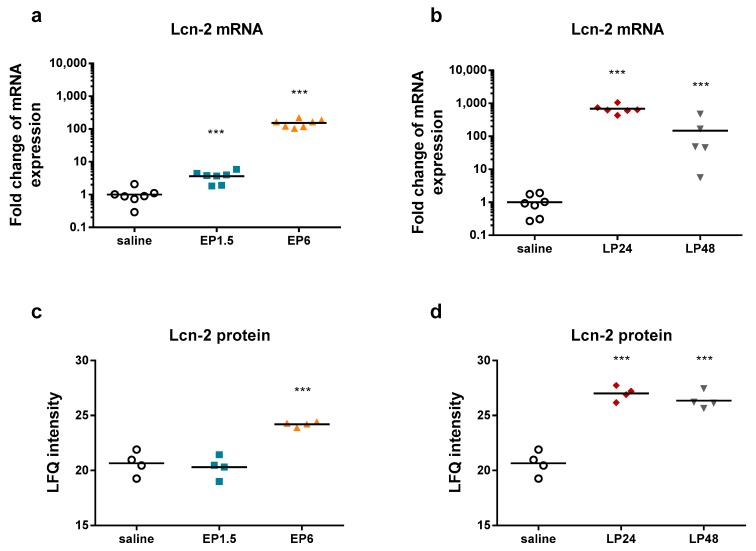
Lipocalin-2 mRNA and protein expression relative to saline-treated control kidneys in mice after LPS administration. ***: *p* ≤ 0.001. (**a**) Fold changes of Lcn-2 mRNA in EP1.5 and EP6 groups. (**b**) Fold changes of Lcn-2 mRNA in LP24 and LP48 groups. (**c**) Label-free quantification (LFQ) intensity values (relative amount) of Lcn-2 protein determined by mass spectrometry in EP 1.5 and EP6 groups. (**d**) LFQ intensity values of Lcn-2 protein determined by mass spectrometry in LP24 and LP48 groups.

**Figure 3:**
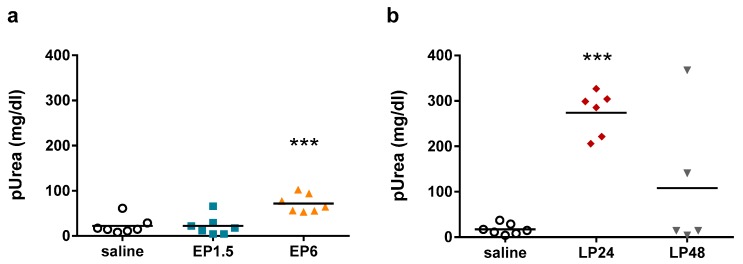
Plasma urea levels in saline- and LPS-treated mice. ***: *p* ≤ 0.001. (**a**) EP1.5 and EP6 groups. (**b**) LP24 and LP48 groups.

LPS significantly upregulated renal Lcn-2 mRNA and protein expression already from 1.5 h and from 6 h, respectively (Figure 2), indicating tubular injury. Plasma urea concentrations were elevated first at 6 h after LPS administration, increased further at 24 h despite the lower LPS dose and started to decrease at 48 h (Figure 3), indicating impaired renal function.

The decrease in both Lcn-2 mRNA and plasma urea concentrations at 48 h indicate reversibility of septic AKI in our experimental setting.

### 2.3. Renal Protein Concentration Changes at Early Phases of AKI

**Figure 4:**
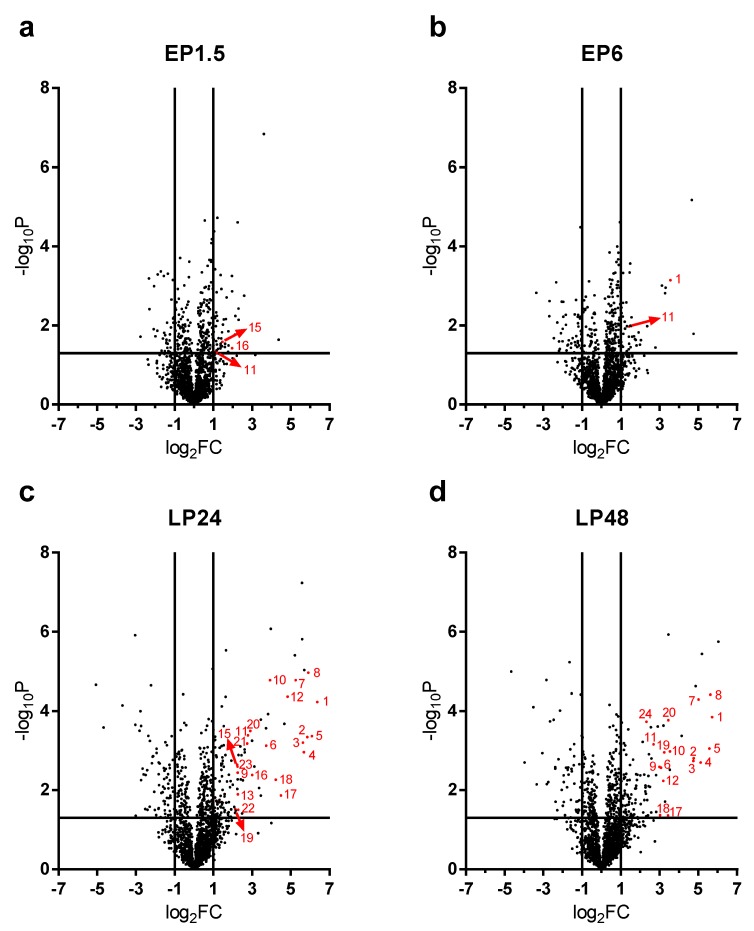
Renal proteome changes after LPS administration. The level of significance (given as –log_10_p values) is plotted against the fold changes (given as log_2_FC). Vertical lines mark 2x fold changes, while horizontal lines mark the significance level of 0.05. λ: APPs (1: Lcn-2, 2: fibrinogen-α, 3: fibrinogen-β, 4: fibrinogen-γ, 5: complement C3, 6: ceruloplasmin, 7: haptoglobin, 8: hemopexin, 9: serum amyloid A-1, 10: serum amyloid A-2, 11: ferritin heavy chain, 12: inter alpha-trypsin inhibitor, heavy chain 4, 13: transferrin, 14: serum albumin, 15: alpha-1-antitrypsin 1-3 and 1-1, 16: serine protease inhibitor A3K, 17: alpha-2-macroglobulin, 18: apolipoprotein A1, 19: alpha-1-acid glycoprotein, 20: beta-2-microglobulin, 21: serine protease inhibitor A3N, 22: apolipoprotein E, 23: vitamin D-binding protein, 24: von Willebrand factor A domain-containing protein 5A). (**a**) EP1.5, (**b**) EP6, (**c**) LP24, (**d**) LP48.

LPS-induced renal proteome effects became highly significant and abundant, involving many proteins first at 24 h after LPS injection (Figure 4c). Only 10-10 proteins were upregulated at least 4-fold (log_2_FC = 2) in the EP1.5 and EP6 groups (Figure 4a,b) and the changes were smaller in the EP than LP groups. Several proteins were upregulated at both time points (Table 1, grey highlights) with quite similar fold changes and rankings. In EP, only Lcn-2 was upregulated more than 4-fold out of the APPs.

### 2.4. Acute Phase Proteins Were the Most Upregulated Proteins in the Kidney in the Late Phase After LPS Administration

As opposed to the moderate proteome changes observed in EP, forty-seven and forty-four proteins were upregulated at least 4× (log_2_FC = 2) at LP24 and LP48, respectively. The first 20 hits are shown in Table 2, the remaining 27 and 24 hits are shown in Supplementary Table S1. Just as in EP, about 2/3 of the proteins were upregulated at both time points, and several proteins were upregulated in both LP and EP.

APPs were abundantly present among the significantly upregulated proteins in LP (Figure 4c,d). At LP24 forty-seven %, while at LP48 thirty-nine % of the identified proteins were APPs. Of the 20 top hits, 12-12 proteins were APPs at LP24 and LP48 (Table 2, bold highlight).

As the top 20 hits included APPs in such a high proportion at LP we reanalyzed the MS data of APPs and collected all the significantly upregulated APPs in Table 3. The list is led by the 3 chains of fibrinogen and complement C3, followed by transport proteins (e.g., ceruloplasmin, haptoglobin, hemopexin, transferrin, ferritin heavy chain) and amyloids, protease inhibitors (inter alpha-trypsin inhibitors, alpha-2-macroglobulin) and serine protease inhibitors (serpins).

Lipocalin-2 (an established AKI marker) appeared third, first and second on the list of the upregulated proteins at EP6, LP24 and LP48, respectively.

The following APPs were enriched in the kidneys after LPS administration in both LP groups: complement C3, fibrinogen-α, -β, -γ, two isoforms of serum amyloid A (Saa1 and Saa2), ceruloplasmin, haptoglobin, hemopexin, inter alpha-trypsin inhibitor heavy chain 4, transferrin, serine protease inhibitor A3K (Serpina3k), alpha-2-macroglobulin (A2m) and beta-2-microglobulin (B2m). Apolipoproteins A1 (ApoA1) and E (ApoE), inter-alpha-trypsin inhibitor heavy chain H1 (Itih1), vitamin D-binding protein or Gc-globulin (DBP) and serine protease inhibitor A3N (Serpina3n) were significantly elevated only in LP24, while alpha-1-acid glycoprotein (A1AGP) and von Willebrand factor A domain-containing protein 5A (Vwa5a) were upregulated only in LP48.

Renal ferritin heavy chain protein was significantly elevated at all 4 time points. Alpha-1-antitrypsin (Serpina1) protein was significantly elevated in EP1.5, LP24 and LP48. Albumin protein fluctuated, it was significantly increased in the EP1.5 and LP24 groups, while it was at control level in EP6 and LP48.

### 2.5. Most of the Upregulated Proteins Were Related to Inflammation in the Late Phase

In addition to the APPs, most of the other proteins upregulated at least 4× in LP were also involved in stress responses based on Gene Ontology (GO) analysis (Table 4). At LP24 sixty % and at LP48 fifty-six % of the top ranking proteins belonged to the ‘response to stress’ category. Among the top 20 hits (Table 2) almost all proteins were either APPs or proteins involved in stress response (highlighted in *italics*). Definitions of the biological process categories are on the GO website (http://www.informatics.jax.org/vocab/gene_ontology/).

### 2.6. Acute Phase Protein Synthesis Was Stimulated in the Kidney after LPS

**Figure 5:**
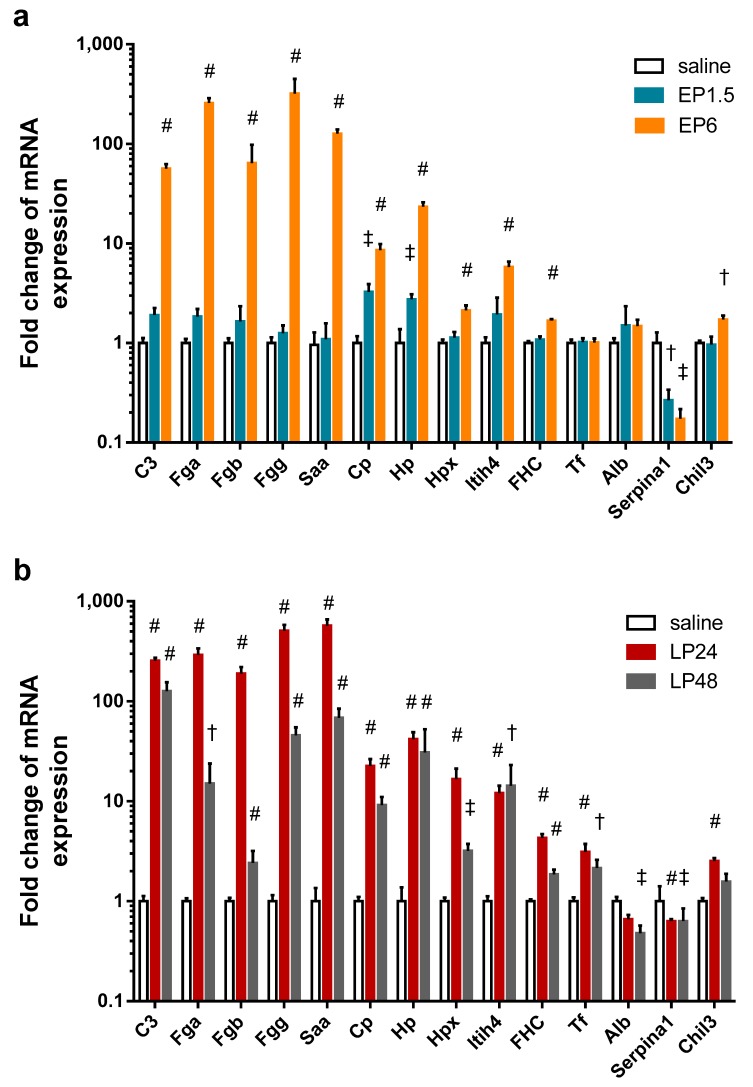
Fold changes of APP mRNA relative to the respective control kidneys in mice after LPS administration. †: *p* < 0.05, ‡: *p* ≤ 0.01, #: *p* ≤ 0.001. C3: complement C3, Fga: fibrinogen-α, Fgb: fibrinogen-β, Fgg: fibrinogen-γ, Saa: serum amyloid A, Cp: ceruloplasmin, Hp: haptoglobin, Hpx: hemopexin, Itih4: inter alpha-trypsin inhibitor heavy chain 4, FHC: ferritin heavy chain, Tf: transferrin, Alb: serum albumin, Serpina1: alpha-1-antitrypsin, Chil3: chitinase-like protein 3. (**a**) EP1.5, EP6. (**b**) LP24, LP48.

To verify the source of the APPs found in the kidney with MS, we conducted quantitative PCR analysis and detected that LPS significantly upregulated the renal mRNA expression of several APPs at EP6 and LP (Figure 5): complement C3, fibrinogen-α, -β, -γ, serum amyloid A, ceruloplasmin, haptoglobin, hemopexin, inter alpha-trypsin inhibitor heavy chain 4, and ferritin heavy chain. Only ceruloplasmin and haptoglobin mRNA were elevated already at 1.5 h. Transferrin expression was upregulated in the kidneys only in LP.

Fibrinogen-α, -β, -γ, serum amyloid A, ceruloplasmin, hemopexin and ferritin heavy chain mRNA decreased at 48 h. Although complement C3 and transferrin mRNA also showed a decreasing tendency in the LP48 group, the difference was not statistically significant.

Albumin expression was unchanged in the kidneys during EP, but showed a decreasing tendency in LP24 with significant downregulation at 48 h.

As the MS analysis did not differentiate between two isoforms of alpha-1-antitrypsin (Serpina1a and Serpina1c), we measured the mRNA expression of these two isoforms separately with primers amplifying only one of them. Neither isoform was expressed in any of the kidney samples. As Zager et al. [32] could amplify alpha-1-antitrypsin in renal tissues, using their primers we were also able to amplify alpha-1-antitrypsin mRNA, however, it was downregulated in all LPS-administered groups. As this primer also amplifies the 1d isoform in addition to 1a and 1c isoforms, the decreased mRNA expression could be attributed to the Serpina1d isoform, not detected by the MS.

Serine protease inhibitor A3K mRNA was undetectable in all kidney samples.

Another inflammatory marker, chitinase-like protein 3 mRNA was upregulated in the EP6 and LP24 groups and still upregulated, but to a less extent, at 48 h.

## 3. Discussion

APP production is a well-known consequence of inflammatory stimuli such as sepsis or LPS. However, the general view is that the main source of plasma APPs is the liver [8,33]. Although production of some APPs in the kidney have been demonstrated before, this is the first comprehensive demonstration of a massive, combined renal APR in sepsis.

The LPS-induced AKI was verified by upregulated TNF-α, IL-6, and Lcn-2 mRNA expression in the kidneys. IL-6 and Lcn-2 mRNA started to decline together with several other factors at 48 h after endotoxin administration, indicating the start of recovery.

The fact that several proteins were included on at least 2 lists (both EP and LP) with quite similar fold changes and rankings supports the reliability of our MS analysis.

Lipocalin-2 (also called as neutrophil gelatinase associated lipocalin: NGAL) is a well-known marker of AKI [34]. It appeared among the upregulated proteins first on the 6-h list and ranked first on the LP24 and second on the LP48 lists, supporting the diagnostic value of Lcn-2 as an AKI marker.

The majority of the mostly upregulated proteins was related to stress and inflammation. For example, the interferon response is a well-known mechanism activated by viral and bacterial infections [35,36]. Correspondingly, interferon-inducible proteins appeared together with APPs in the LP lists.

Regarding the renal APR, our results are also in line with scarce previous findings demonstrating that endotoxin upregulated renal mRNA and/or protein expression of haptoglobin [26,27,28], hemopexin [29], ceruloplasmin [23], serum amyloid A [23,27], complement C3 [24,25], and all three chains of fibrinogen [22] 24 h after LPS administration, while albumin was downregulated [31]. We identified additional APPs upregulated by LPS in the kidney such as inter alpha-trypsin inhibitor heavy chain 4, transferrin, ferritin heavy chain, alpha-2-macroglobulin, apolipoprotein A1, apolipoprotein E, vitamin D-binding protein, inter-alpha-trypsin inhibitor heavy chain H1, serine protease inhibitor A3K, A3N, and alpha-1-antitrypsin. Although transferrin is a negative APP in humans, it is regarded as a positive APP in mice [37].

The identified highly upregulated APPs (coagulation system components, complement C3, transport proteins, amyloids and protease inhibitors) have been considered to be primarily produced by the liver [33]. However, several APPs have been demonstrated to be produced in other organs under physiological conditions. Examples include haptoglobin expression in the skin and lung [8], transferrin in the brain [38], ceruloplasmin in the kidney, mammary gland and brain [39], hemopexin in the nervous system and retina [40], complement C3 in the immune cells and the kidney [41], and serum amyloid A in many tissues including the kidney [42]. Ferritin is expressed in all organs including the kidneys [43]. In general, APPs are expressed at a low level in extrahepatic tissues under physiologic conditions but can be induced by local injury [8].

Chitinase-like protein 3 (Chil3) was also upregulated in the kidney. It is a rodent-specific member of the chitinase-like protein family. It is a chemoattractant for eosinophils and a marker of murine M2 macrophages and N2 neutrophils [44,45,46,47]. Another, highly homologous member of the chitinase-like protein family, chitinase-3-like protein 1 (CHI3L1) is an APP in humans [48]. Both CHI3L1 and Chil3 were elevated in the serum of septic mice [49], suggesting that Chil3 may also be involved in the APR.

The role of this endotoxin-associated renal APR is probably homeostatic, by providing tissue protection [7]. Some APPs are effectors of the immune system (e.g., complement C3 [50]), some recruit immune cells (e.g., fibrinogen [51], serum amyloid A [52] and chitinase-like protein 3 [44]), some have antioxidant or anti-inflammatory functions (e.g., ceruloplasmin [53], hemopexin [54], haptoglobin [55], inter alpha-trypsin inhibitor heavy chain 4 [56], alpha-1-acid glycoprotein [57], and apolipoproteins [58,59]), some protect tissues from proteolytic destruction (e.g., alpha-1 antitrypsin [60] and alpha-2-macroglobulin [61]), some have cytoprotective scavenger functions (e.g., ferritin heavy chain [62] and transferrin [63] bind free Fe, haptoglobin sequesters hemoglobin [55], hemopexin binds heme and bivalent metal ions [54], apolipoprotein A1 [58] and vitamin D-binding protein [64] neutralize LPS), and some initiate tissue repair (e.g., fibrinogen [50]).

A likely role of the APPs produced in the kidney is mediating cell survival [65]. For example, the heme-hemopexin complex [54] and ferritin heavy chain [66] have been shown to inhibit apoptosis. The role of ferritin heavy chain has already been studied in different AKI settings. Rhabdomyolysis, cisplatin-induced AKI and unilateral ureteral obstruction all had worse outcomes in proximal tubule-specific ferritin heavy chain knockout mice [67,68]. Furthermore, overexpression of ferritin heavy chain in the kidneys prior to ischemia-reperfusion injury had cytoprotective effects [69]. It has also been demonstrated that sublethal doses of LPS can protect the kidneys from a subsequent lethal damage such as severe ischemia-reperfusion injury [70,71]. If APPs indeed provide protection to the kidney, they may be functionally involved in the mechanism of LPS-induced preconditioning.

Although most APPs serve to preserve tissue integrity, some of them can mediate both protection and injury. For example, fibrinogen can enhance inflammation by activating immune cells, while it initiates tissue repair as a scaffold for tissue reconstruction [51,72,73]. In fibrinogen knockout mice, renal fibrogenesis was reduced after unilateral ureteral obstruction [74], but renal function and survival was worse following ischemia-reperfusion injury [75].

Serum amyloid A also mediates both proinflammatory and anti-inflammatory processes. It can attract phagocytes and enhance proinflammatory cytokine production but can promote anti-inflammatory cytokine production and polarisation of macrophages to M2 phenotype [52,76]. Similarly, ceruloplasmin is also known to have both prooxidant and antioxidant activities [53].

The markedly enhanced renal production of a great number of APPs puts them forward as biomarker candidates. Zager et al. [28] found that LPS induced greater upregulation of haptoglobin in the kidneys than in the liver in mice, and they detected elevated urinary haptoglobin in patients with AKI. Also, chitinase-like proteins, including Chil3 were suggested to be potential biomarkers of septic AKI in both mice and human [49].

However, before any APP is considered as a biomarker of septic AKI, their timely expression pattern should be determined. Most previous studies determined changes in APPs solely 24 h after endotoxin administration. Only scarce previous findings are available regarding EP, e.g., haptoglobin was upregulated [28], while albumin was unchanged [31], and hemopexin was also slightly increased 4 h after LPS administration in mice [29].

Taken together, renal gene expression of many APPs was induced already during the EP and peaked at 24 h. Among the APPs studied, ceruloplasmin, haptoglobin, and Lcn-2 mRNA were upregulated earliest. They were followed at 6 h by complement C3, fibrinogen-α, -β, -γ, hemopexin, serum amyloid A, inter alpha-trypsin inhibitor heavy chain 4, and ferritin heavy chain. All these gene expression changes in EP translated into elevated protein levels in the kidneys at the established phase at 24 h. Only ferritin heavy chain protein was already elevated in EP. Transferrin responses were delayed as both mRNA and protein appeared elevated first at LP24. Reduction of the mRNA expression of fibrinogen-α, -β, -γ, serum amyloid A, ceruloplasmin, hemopexin, and ferritin heavy chain by 48 h vs. 24 h, suggests initiation of recovery.

As a negative APP, renal albumin mRNA was suppressed by LPS during LP. The increase in renal serine protease inhibitor A3K and alpha-1-antitrypsin isoforms Serpina1a and 1c were not the result of renal expression. We hypothesize that their source may be the liver, since serine protease inhibitor A3K is a liver-specific gene [77]. Based on our data, none of the kidney specific APPs are earlier markers of septic AKI than Lcn-2.

## 4. Materials and Methods

### 4.1. Animal Studies

Male Naval Medical Research Institute (NMRI, Bethesda, MD, USA) mice (Hsd:Win:NMRI mice (RRID_MGI_MGI:6198565); Toxi-Coop Ltd., Budapest, Hungary) weighing 25–30 g were housed under standard conditions with free access to food and tap water. All protocols were approved by the Pest County Government Office and the Animal Ethics Committee of Semmelweis University (code: PE/EA/2202-5, date: 14 December 2017).

### 4.2. LPS Application

Mice were injected with LPS (0111:B4; Sigma-Aldrich, Budapest, Hungary) at the doses of 10 or 40 mg/kg bodyweight (BW) intraperitoneally (i.p.). These maximal doses were selected based on our previous experiments to ensure septic shock but no lethality throughout the follow-up period [70,71]. Two time points represented the early phase (EP, 1.5 and 6 h), while the other two represented the late phase (LP) of LPS-induced inflammation and AKI (24 and 48 h) [78]. LPS was freshly suspended in sterile saline immediately before use. Animals were divided into four treatment groups as follows:

- Group 1 (EP1.5): LPS at 40 mg/kg BW, and sacrificed at 1.5 h post-injection (*n* = 7)
- Group 2 (EP6): LPS at 40 mg/kg BW, and sacrificed at 6 h post-injection (*n* = 7)
- Group 3 (LP24): LPS at 10 mg/kg BW, and sacrificed at 24 h post-injection (*n* = 7)
- Group 4 (LP48): LPS at 10 mg/kg BW, and sacrificed at 48 h post-injection (*n* = 7)

Mice (*n* = 7/each dose) received equal volumes of saline to serve as controls.

### 4.3. Organ Harvest

Mice were injected with 5000 U/kg BW heparin i.p. (Ratiopharm GmbH, Ulm, Germany) and 3 min later they were sacrificed by cervical dislocation. The chest was opened and blood was collected from the thoracic cavity after cross-section of the vena cava. Blood was removed from parenchymal organs and the whole circulation by injecting 10 mL 4 °C saline transcardially. The kidneys were removed, decapsulated and cut into pieces. One piece from each kidney was placed in 500 µL TRI Reagent (TR 118, Molecular Research Center, Inc., Cincinnati, OH, USA). Together with the parts of the kidney were snap frozen in liquid nitrogen and kept at −80 °C for RNA isolation and proteomic analysis.

Livers were also collected similarly, from intact NMRI mice for control purposes.

### 4.4. Plasma Urea Determination

Plasma urea concentrations were measured by a urease and glutamate-dehydrogenase enzymatic assay with colorimetric detection according to the manufacturer’s protocol (Diagnosticum Zrt., Budapest, Hungary). The urea concentration of the samples was determined using a standard curve.

### 4.5. Tissue Homogenization

Four kidney samples selected as most representative from each treatment group and four control kidney samples were processed for mass spectrometry analysis as described before [79] with a slightly modified protocol. Frozen tissues were cut into pieces of about 30 mg on dry ice to prevent thawing. Next, 8 µL lysis buffer/mg of tissue was added to each piece. The lysis buffer contained 50 mM Tris pH 8.0 (SERVA Electrophoresis GmbH, Heidelberg, Germany), 150 mM NaCl (Thermo Fisher Scientific, San Jose, CA, USA), 1 mM EDTA (SERVA Electrophoresis GmbH, Heidelberg, Germany), 1% NP-40 lysis buffer (Roche, Basel, Switzerland), 0.5% Na-deoxycholate (Sigma-Aldrich, Maribor, Slovenia), 0.1% sodium dodecyl sulfate (Pierce Chemical, Dallas, TX, USA) and 1% protease inhibitor cocktail (Sigma-Aldrich, Maribor, Slovenia). The tissue was homogenized by Dounce homogenizer, and then repeatedly passed through a 0.7 × 38 mm syringe needle for 20 times for further homogenization. The samples were then gently shaken for 30 min on ice (Rotamax 120, Heidolph Instruments GmbH, Schwabach, Germany). After centrifugation (16,000 *g*, 10 min), the protein concentration in the supernatant was determined by the Bradford assay (Bio-Rad Laboratories, Inc., Hercules, CA, USA) and 150 µg of total protein from each sample was incubated with loading buffer (10 mM Tris pH 6.8; SERVA Electrophoresis GmbH, Heidelberg, Germany); 7.8% glycerol (CARLO ERBA Reagents, Val de Reuil, France); 0.2% SDS (Pierce Chemical, Dallas, TX, USA); 0.01% Bromphenol blue (Sigma-Aldrich, Maribor, Slovenia)) containing 10 mM dithiothreitol (DTT) (Fluka Biochemica, Steinheim, Germany) at 95 °C for 5 min. The samples were stored at −20 °C until further processing.

### 4.6. Sample Preparation for Mass Spectrometry

Sample preparation for LC-MS/MS analysis was performed as described previously [80]. Briefly, 150 µg of total protein from each sample was separated on a 12.5% SDS-PAGE gel (Lonza, Basel, Switzerland). The gel was stained with Coomassie brilliant blue and each of the protein lanes was cut into six bands and destained with 25 mM ammonium bicarbonate in 50% acetonitrile. The gel pieces were washed with acetonitrile, vacuum dried and rehydrated in reducing solution (10 mM DTT; Fluka Biochemica, Steinheim, Germany), 25 mM NH_4_HCO_3_. After 45-min incubation at 56 °C, solution was exchanged to alkylating solution (55 mM iodoacetamide; Amersham Biosciences, Little Chalfont, UK), 25 mM NH_4_HCO_3_ and samples were incubated in the dark at room temperature for 30 min. The gel pieces were washed with acetonitrile and vacuum dried before rehydrating in 80 µL of trypsinization buffer (25 mM NH_4_HCO_3_) containing 1 µg of sequencing-grade modified porcine trypsine (Promega, Madison, WI, USA) per sample. After overnight incubation at 37 °C, the trypsin solution was collected and remaining peptides were extracted from the gel pieces using extraction solution (50% acetonitrile, 5% formic acid; JT Baker, Center Valley, PA, USA). Trypsin solution was added to extraction solution and concentrated by vacuum drying to a final volume of about 20 µL.

### 4.7. Mass Spectrometry Analysis

LC-MS/MS analysis was performed with an EASY-nanoLC II HPLC unit (Thermo Fisher Scientific, Waltham, MA, USA) coupled to an Orbitrap LTQ Velos mass spectrometer (Thermo Fisher Scientific, Waltham, MA, USA). Samples containing 0.1% FA were loaded onto a C18 trapping column (Proxeon Easy-column, Thermo Fischer Scientific, West Palm Beach, FL, USA) and separated on a C18 PicoFrit Aquasil analytical column (New Objective, Inc., Woburn, MA, USA). The peptides were eluted using a 5–40% (v/v) 90 min linear gradient of acetonitrile in a 0.1% formic acid solution at a constant flow rate of 300 nL/min. The full MS mass spectra were acquired with the Orbitrap mass analyzer in the mass range of 300 to 2000 m/z at resolution of 30,000. The MS/MS spectra were obtained by HCD fragmentation of the nine most intense MS precursor ions and recorded at a resolution of 7500. Only the precursor ions with assigned charge states (> 1) were selected for MS/MS fragmentation. The dynamic exclusion was set to repeat count of 1, repeat duration of 30 s, and exclusion duration of 20 s.

### 4.8. Data Analysis

Data analysis was performed as described previously [80] with minor modifications. MaxQuant proteomics software (version 1.6.0.13; Max-Planck Institute for Biochemistry, Martinsried, Germany) was used for database search and quantification by spectral counting [81]. Database search was performed against a Mus musculus Uniprot database (database date 15.10.2017, 16,923 entries). For the database searches methionine oxidation (+15.995 Da) and protein N-terminal acetylation (+45.011 Da) were set as variable modifications. Carbamidomethylation of cysteines (+57.021 Da) was set as fixed modification. Trypsin cleavage after arginine and lysine was used as the enzyme specificity. For database searches one missed cleavage was allowed. In addition, precursor ion and fragment ion mass tolerances were set to 20 ppm and 0.5 Da, respectively. A reversed database search was performed and the false discovery rate was set at 1% for peptide and protein identifications. Raw data and database search files are available via ProteomeXchange with identifier PXD014664 [82]. Relative quantification of identified proteins was performed by label-free quantification (LFQ) algorithm in MaxQuant.

### 4.9. RNA Preparation

Total RNA was extracted from the kidneys and livers with TRI Reagent (TR 118, Molecular Research Center, Inc., Cincinnati, OH, USA) according to the protocol provided by the manufacturer. RNA concentration and purity was checked with NanoDrop 2000 c spectrophotometer (Thermo Fisher Scientific, Waltham, MA, USA) and RNA integrity was verified by electrophoresing the samples on 1% agarose gel (Invitrogen Ltd., Paisley, UK). The RNA solutions were stored at −80 °C until further procedures.

### 4.10. qPCR Analysis of Gene Expression

The gene expression of MS identified APPs assessed by qPCR are summarized in Table 5. Each primer was tested on normal liver samples as positive control. TNF-α and IL-6 mRNA levels were determined to assess inflammation induced by LPS administration in the kidneys of mice. Renal tubular damage was assessed based on Lcn-2 gene expression. The endogenous reference gene was GAPDH.

Messenger RNA (mRNA) levels were measured as described previously [10]. Reverse transcription into cDNA was carried out by the High-Capacity cDNA Archive Kit (Applied Biosystems™, Foster City, CA, USA) according to the manufacturer’s protocol. In brief, 1 μg of total RNA was denaturized at 70 °C for 5 min. After the annealing of the random hexamer primers on the RNA template at 25 °C for 10 min, cDNA was synthesized at 37 °C for 2 h. The reaction was terminated by heat inactivation (85 °C for 2 min). Gene expression from kidney tissue homogenates was evaluated on the Bio-Rad C1000™ Thermal Cycler with CFX96™ Optics Module real-time PCR system (Bio-Rad Laboratories, Inc., Hercules, CA, USA). The PCR reaction was performed with SensiFAST™ SYBR No-ROX Kit (Bioline Reagents Limited, London, UK), according to the manufacturer’s protocol. Primers (Table 5) were designed by NCBI/Primer-BLAST online software with the exception of the Serpina1 primer that was adapted from Zager et al. [32] Primers were synthesized by Integrated DNA Technologies. Primer annealing was set to 60 °C. All samples were measured in duplicates and expression was calculated using the relative quantification (ΔΔ*C*_q_) method. The efficiency of the qPCR reaction was verified with standard curves. The melting curve was also viewed to detect any abnormality of the PCR product. Expression was considered to be below the limit of detection in the samples in which no specific amplification product was present.

One mouse in the LP48 group was excluded from the statistical analysis because most of its mRNA expression values were identified as outliers within the group (ROUT method).

### 4.11. Protein Classification

Gene Ontology Enrichment Analysis powered by PANTHER [83,84,85,86] was performed to assign proteins to gene ontology biological function categories. Proteins were classified as APPs based on the comprehensive list of classical and putative APPs by Salgado et al. [87].

### 4.12. Statistical Analysis

All continuous data are expressed as mean ± standard error of the mean (SEM), unless otherwise stated. LFQ intensity values were log2 transformed for statistical analysis. Fold changes (FC) were determined as the difference of the means between the treated and control groups (log_2_FC). Messenger RNA fold changes were calculated by dividing each normalized expression value with the mean of the respective control group and values were log10 transformed for statistical analysis. ROUT method [88] (combines a robust nonlinear regression with an outlier identification method based on the false discovery rate) was used to detect outliers. One-way analysis of variance (ANOVA) followed by Tukey’s multiple comparisons test was performed for between-group comparisons. If Bartlett’s test indicated heterogeneity of variances, the Kruskal–Wallis one-way analysis of variance by ranks was performed followed by Dunn’s test. If there were only two groups, unpaired Student’s t-test was used for comparison. The null hypothesis was rejected if *p* < 0.05. Statistical analysis was performed using GraphPad Prism6 (GraphPad Software Inc., San Diego, CA, USA).

## 5. Conclusions

In conclusion, a massive local acute phase response was induced during endotoxemia-associated AKI. Most acute phase proteins (APPs) were upregulated already in the early phase (EP), in some cases as early as 1.5 h after endotoxin administration. Their altered expression peaked at 24 h and some of them started to recover by 48 h. The upregulated mRNA initiated in EP translated into elevated renal protein levels by 24 h. Although a few APPs have been identified in the kidneys in endotoxemia before, such a broad extent of the LPS-induced local, renal APR has not been recognized before.

## Figures and Tables

**Table 1:** Proteins significantly upregulated at least 4-fold (log_2_FC = 2) relative to the saline-injected control kidneys in EP in mice. Acute phase proteins (APPs) are highlighted in bold, proteins present at both time points are highlighted in grey. log_2_FC: log2 transformed values of fold change.

	EP1.5	log_2_FC	EP6	log_2_FC
1	GTPase HRas	4.36	GTPase HRas	4.75
2	Chitinase-like protein 3	3.60	Chitinase-like protein 3	4.66
3	Protein S100-A9	2.59	**Lipocalin-2**	3.55
4	Succinate-semialdehyde dehydrogenase, mitochondrial	2.30	H-2 class I histocompatibility antigen, L-D alpha chain	3.31
5	Mesencephalic astrocyte-derived neurotrophic factor	2.29	CD151 antigen	3.28
6	CD151 antigen	2.27	Protein S100-A9	3.12
7	S-adenosylmethionine synthase isoform type-2	2.26	Major urinary protein 3	2.78
8	Proteasome subunit alpha type-7	2.20	Transmembrane emp24 domain-containing protein 2	2.36
9	Peptidyl-prolyl cis-trans isomerase FKBP3	2.13	Proteasome subunit alpha type-7	2.20
10	Transmembrane emp24 domain-containing protein 2	2.10	Cytochrome c oxidase subunit 7C, mitochondrial	2.04

**Table 2:** List of top 20 proteins significantly upregulated at least 4-fold (log_2_FC = 2) relative to the controls kidneys at LP in mice. APPs are highlighted in bold, other proteins involved in response to stress are highlighted in italics, proteins present at both time points are highlighted in grey. log_2_FC: log2 transformed values of fold change.

	LP24	log_2_FC	LP48	log_2_FC
1	**Lipocalin-2**	6.36	*H-2 class I histocompatibility antigen, L-D alpha chain*	6.03
2	**Complement C3**	6.09	**Lipocalin-2**	5.71
3	**Hemopexin**	5.90	**Hemopexin**	5.62
4	**Fibrinogen alpha chain**	5.85	**Complement C3**	5.58
5	*H-2 class I histocompatibility antigen, L-D alpha chain*	5.69	*Interferon-inducible GTPase 1*	5.19
6	**Fibrinogen gamma chain**	5.67	**Fibrinogen gamma chain**	5.12
7	**Fibrinogen beta chain**	5.62	**Haptoglobin**	5.01
8	*Interferon-inducible GTPase 1*	5.59	*Interferon-induced guanylate-binding protein 2*	4.86
9	*Interferon-induced guanylate-binding protein 2*	5.57	**Fibrinogen alpha chain**	4.76
10	**Haptoglobin**	5.25	**Fibrinogen beta chain**	4.75
11	*T cell specific GTPase 1*	5.21	*Immunity-related GTPase family M protein 1*	4.14
12	**Inter alpha-trypsin inhibitor, heavy chain 4**	4.83	**Serum amyloid A-2 protein**	3.54
13	*Immunity-related GTPase family M protein 1*	4.66	Major vault protein	3.52
14	**Serine protease inhibitor A3K**	4.49	*Chitinase-like protein 3*	3.46
15	**Alpha-2-macroglobulin**	4.21	**Beta-2-microglobulin**	3.45
16	*Chitinase-like protein 3*	3.96	**Serine protease inhibitor A3K**	3.44
17	**Serum amyloid A-2 protein**	3.92	*T cell specific GTPase 1*	3.28
18	*CD151 antigen*	3.82	**Alpha-1-acid glycoprotein 1, Alpha-1-acid glycoprotein 2**	3.24
19	**Ceruloplasmin**	3.72	Napsin-A	3.21
20	Major vault protein	3.72	**Inter alpha-trypsin inhibitor, heavy chain 4**	3.19

**Table 3:** Log2 transformed label-free quantification (LFQ) intensity values of APPs determined by mass spectrometry in the kidneys of mice after LPS administration. Values are given as mean ± standard deviation (SD). *: *p* < 0.05, **: *p* ≤ 0.01, ***: *p* ≤ 0.001. EP (1.5 and 6 h) and LP (24 and 48 h) were statistically analysed separately (double line). Lcn-2: lipocalin-2, C3: complement C3, Fga: fibrinogen-α, Fgb: fibrinogen-β, Fgg: fibrinogen-γ, Saa: serum amyloid A, Cp: ceruloplasmin, Hp: haptoglobin, Hpx: hemopexin, Itih4: inter alpha-trypsin inhibitor heavy chain 4, FHC: ferritin heavy chain, Tf: transferrin, Alb: serum albumin, Serpina1: alpha-1-antitrypsin, Serpina3k: serine protease inhibitor A3K, Serpina3n: serine protease inhibitor A3N, A2m: alpha-2-macroglobulin, B2m: beta-2-microglobulin, ApoA1: apolipoprotein A1, ApoE: apolipoprotein E, A1AGP: α-1-acid glycoprotein, Itih1: inter alpha-trypsin inhibitor heavy chain 1, DBP: vitamin D-binding protein, Vwa5a: von Willebrand factor A domain-containing protein 5A.

	Saline	EP1.5	EP6	LP24	LP48
Lcn-2	20.65 ± 1.10	20.31 ± 1.00	24.20 ± 0.23 ***	27.01 ± 0.66 ***	26.36 ± 0.77 ***
C3	21.94 ± 1.69	23.29 ± 1.23	21.24 ± 1.20	28.03 ± 0.41 ***	27.51 ± 0.69 ***
Fga	21.78 ± 1.61	23.20 ± 2.08	22.85 ± 1.77	27.63 ± 0.53 ***	26.54 ± 0.67 ***
Fgb	21.53 ± 1.56	21.84 ± 0.85	21.36 ± 0.55	27.15 ± 0.74 ***	26.28 ± 0.88 ***
Fgg	21.26 ± 1.89	22.06 ± 1.42	23.00 ± 1.58	26.93 ± 0.44 ***	26.38 ± 0.55 ***
Saa1	20.84 ± 0.79	21.03 ± 0.79	21.01 ± 0.59	23.09 ± 0.57 **	23.84 ± 0.92 ***
Saa2	20.38 ± 0.50	19.77 ± 0.78	21.28 ± 0.34	24.30 ± 0.39 ***	23.91 ± 1.09 ***
Cp	21.34 ± 1.15	20.52 ± 0.68	19.85 ± 0.79	25.06 ± 0.29 ***	24.41 ± 0.50 ***
Hp	21.06 ± 0.62	20.53 ± 0.48	21.04 ± 1.10	26.31 ± 0.58 ***	26.07 ± 0.76 ***
Hpx	22.15 ± 0.84	20.66 ± 0.37	21.91 ± 1.03	28.05 ± 0.27 ***	27.77 ± 0.67 ***
Itih4	20.77 ± 0.76	20.73 ± 0.92	21.14 ± 0.49	25.60 ± 0.51 ***	23.96 ± 1.33 **
FHC	23.57 ± 0.72	24.65 ± 0.47 *	24.99 ± 0.28 **	26.36 ± 0.32 ***	26.26 ± 0.43 ***
Tf	26.25 ± 1.26	27.36 ± 0.44	26.83 ± 0.46	28.51 ± 0.27 **	27.79 ± 0.47 *
Alb	29.60 ± 0.85	31.06 ± 0.51 *	29.97 ± 0.29	31.82 ± 0.20 ***	30.47 ± 0.50
Serpina1a,c	23.84 ± 1.30	25.81 ± 0.70 *	25.44 ± 0.37	26.84 ± 0.28 ***	25.32 ± 0.34 *
Serpina3k	22.88 ± 2.55	26.04 ± 0.79	25.22 ± 0.96	27.37 ± 0.52 **	26.32 ± 0.91 *
Serpina3n	20.53 ± 0.75	20.83 ± 0.84	20.90 ± 0.47	23.27 ± 0.41 **	22.06 ± 1.18
A2m	22.13 ± 1.92	23.45 ± 2.12	21.00 ± 0.52	26.34 ± 0.52 **	25.16 ± 1.37 *
B2m	20.80 ± 0.75	20.05 ± 0.49	21.82 ± 1.45	23.70 ± 0.24 ***	24.25 ± 0.37 ***
ApoA1	25.16 ± 1.35	26.48 ± 0.67	25.26 ± 0.34	27.30 ± 0.68 *	26.22 ± 0.35
ApoE	20.42 ± 1.10	21.58 ± 1.12	20.58 ± 0.95	22.70 ± 1.20 *	21.46 ± 0.81
A1AGP	20.12 ± 0.96	20.25 ± 0.58	20.57 ± 0.56	21.12 ± 1.02	23.36 ± 0.55 ***
Itih1	20.10 ± 0.68	21.14 ± 0.50	21.08 ± 1.02	21.32 ± 0.36 *	21.10 ± 0.69
DBP	20.54 ± 0.75	21.04 ± 0.45	20.46 ± 0.29	22.78 ± 0.50 ***	21.33 ± 0.37
Vwa5a	20.75 ± 0.56	20.56 ± 0.58	20.44 ± 0.69	22.29 ± 1.57	23.08 ± 0.12 *

**Table 4:** Upregulated proteins (log_2_FC ≥ 2) other than APPs were assigned to biological process categories in LP based on the Gene Ontology analysis of proteins, and the top 10 categories are listed.

	GO Biological Process (LP24)	No. of Entities	*p*-Value	GO Biological Process (LP48)	No. of Entities	*p*-Value
1	response to stress	16	9.68 × 10^−8^	response to stress	16	1.88 × 10^−7^
2	immune system process	15	1.22 × 10^−8^	immune system process	15	2.29 × 10^−8^
3	defense response	14	1.07 × 10^−10^	defense response	14	1.96 × 10^−10^
4	response to chemical	13	8.72 × 10^−5^	immune response	13	5.30 × 10^−9^
5	immune response	12	3.84 × 10^−8^	response to organic substance	12	2.26 × 10^−5^
6	response to organic substance	12	1.47 × 10^−5^	cellular response to chemical stimulus	12	1.09 × 10^−5^
7	cellular response to chemical stimulus	12	7.03 × 10^−6^	innate immune response	11	9.75 × 10^−10^
8	innate immune response	11	6.24 × 10^−10^	cellular response to organic substance	11	6.32 × 10^−6^
9	multi-organism process	11	2.76 × 10^−5^	multi-organism process	11	4.06 × 10^−5^
10	response to external stimulus	11	1.36 × 10^−5^	response to external stimulus	11	2.02 × 10^−5^

**Table 5:** Sequences of primers used for measuring the expression of target genes by qPCR. Fga: fibrinogen-α, Fgb: fibrinogen-β, Fgg: fibrinogen-γ, C3: complement C3, Cp: ceruloplasmin, Hp: haptoglobin, Hpx: hemopexin, FHC: ferritin heavy chain, Tf: transferrin, Saa: serum amyloid A, Itih4: inter alpha-trypsin inhibitor heavy chain 4, Alb: serum albumin, Serpina1: alpha-1-antitrypsin, Serpina3k: serine protease inhibitor A3K, and Chil3: chitinase-like protein 3, Lcn-2: lipocalin-2.

Target	Forward Primer	Reverse Primer
Fga	TGAGCCATCCCTAAACGCAG	GCCAGTCTGAGTCCTTGCAT
Fgb	GGCTTCACGGTACAGAACGA	ATTTGGATTGGCTGCATGGC
Fgg	ATGAACAAATGTCACGCAGGC	TCAACTTCATGATCCACGCTGA
C3	ATCCAGACAGACCAGACCATCT	AGGATGACGACTGTCTTGCC
Cp	AGGCCCTGATGAGGAACATCT	TGCTGTGAGGAGCGACCT
Hp	GTGGAGCACTTGGTTCGCTA	CCATAGAGCCACCGATGATGC
Hpx	CGCTACTACTGCTTCCAGGG	AGCTATGCCATCCATCACGG
FHC	CCCTTTGCAACTTCGTCGTTC	GAGCCACATCATCTCGGTCAA
Tf	CCCAAGGATGGACTACAGGC	TGATGCTCCACTCGTCACAC
Saa	GCAGGATGAAGCTACTCACCA	TGGTCAGCAATGGTGTCCTC
Itih4	TCCGTTGCAGCACAATATCCT	TCACTTCGAGCCACGAGAAC
Alb	TTGGCAACAGACCTGACCAA	GTGTCATGCTCCACCTCACT
Serpina1	TTCCAACACCTCCTCCAAAC	CACCGCCTCAGCTATCTTTC
Serpina1a	TCAATTCAGTGTCCTCTCCAGC	AAGTCTCCCAGGTTTGTAGCG
Serpina1c	CCAGAAGGTTAGCCCAGATCCAC	GGCATAGAATAAGGAACGGCTAGT
Serpina3k	CAGCAGGACAGATTCCAGCC	AGGAGTCAGCTATCACAGAGGC
Chil3	AGAAGCTCTCCAGAAGCAATCC	TCAGCTGGTAGGAAGATCCCA
Lcn-2	AGGTGGTACGTTGTGGGC	CTGTACCTGAGGATACCTGTG
TNF-α	AAATGGCCTCCCTCTCATCA	AGATAGCAAATCGGCTGACG
IL-6	GATGCTACCAAACTGGATATAATC	GGTCCTTAGCCACTCCTTCTGTG
GAPDH	CCAGAATGAGGATCCCAGAA	ACCACCTGAAACATGCAACA

## Supplementary Materials

Supplementary materials can be found at https://www.mdpi.com/1422-0067/21/1/200/s1.

## Abbreviations

A1AGP	α-1-acid glycoprotein
A2m	alpha-2-macroglobulin
AKI	acute kidney injury
Alb	serum albumin
ApoA1	apolipoprotein A1
ApoE	apolipoprotein E
APP	acute phase protein
APR	acute phase response/reaction
BW	bodyweight
C3	complement C3
Chil3	chitinase-like protein 3
CHI3L1	Chitinase-3-like protein 1
Cp	ceruloplasmin
DBP	vitamin D-binding protein
EP	early phase (of LPS-induced systemic inflammation)
Fga	fibrinogen-α
Fgb	fibrinogen-β
FC	fold change
Fgg	fibrinogen-γ
FHC	ferritin heavy chain
GO	Gene Ontology
Hp	haptoglobin
Hpx	hemopexin
i.p.	intraperitoneal
Itih1	inter-alpha-trypsin inhibitor heavy chain H1
Itih4	inter alpha-trypsin inhibitor heavy chain 4
LFQ	label free quantification (of mass spectrometry data)
LP	late phase (of LPS-induced systemic inflammation)
LPS	lipopolysaccharide
Lcn-2	lipocalin-2
NGAL	neutrophil gelatinase-associated lipocalin
NMRI	(mice developed by the) Naval Medical Research Institute
PAI-1	plasminogen activator inhibitor-1
Saa	serum amyloid A
Serpina1	alpha-1-antitrypsin
Serpina3k	serine protease inhibitor A3K
Serpina3n	serine protease inhibitor A3N
Tf	transferrin
Vwa5a	von Willebrand factor A domain-containing protein 5A
