# Supplementary material for: The Acute Phase Response Is a Prominent Renal Proteome Change in Sepsis in Mice
Source: Int J Mol Sci. 2019 Dec 27;21(1):200. doi: 10.3390/ijms21010200 (PMC6982205; doi:10.3390/ijms21010200)
Supplement: Supplementary file 1 [file ijms-21-00200-s001.zip › ijms-658764-supplementary.docx]

**Supplementary Table S1.** List of top 21-47 proteins significantly upregulated at least 4-fold (log_2_FC=2) relative to the controls kidneys at LP in mice. APPs are highlighted in bold, other proteins involved in response to stress are highlighted in italics, proteins present at both time points are highlighted in grey. log_2_FC: log2 transformed values of fold change.

|  | **LP24** | **log_2_FC** | **LP48** | **log_2_FC** |
| --- | --- | --- | --- | --- |
| 21 | *Interferon-induced transmembrane protein 3* | 3.45 | **Ceruloplasmin** | 3.07 |
| 22 | Murinoglobulin-1, Murinoglobulin-2 | 3.45 | **Alpha-2-macroglobulin** | 3.03 |
| 23 | Major urinary protein 3 | 3.36 | **Serum amyloid A-1 protein** | 3.00 |
| 24 | Proteasome activator complex subunit 2 | 3.11 | *Interferon-induced transmembrane protein 3* | 2.91 |
| 25 | **Alpha-1-antitrypsin 1-3, Alpha-1-antitrypsin 1-1** | 3.00 | Cytochrome P450 4A10 | 2.83 |
| 26 | *Guanylate-binding protein 4* | 3.00 | Proteasome activator complex subunit 2 | 2.82 |
| 27 | **Beta-2-microglobulin** | 2.90 | **Ferritin heavy chain** | 2.69 |
| 28 | **Ferritin heavy chain** | 2.79 | *Guanylate-binding protein 4* | 2.57 |
| 29 | **Serine protease inhibitor A3N** | 2.74 | Xanthine dehydrogenase/oxidase | 2.55 |
| 30 | *Deoxynucleoside triphosphate triphosphohydrolase SAMHD1* | 2.62 | *H-2 class II histocompatibility antigen, A-Q beta and A-D beta chain* | 2.50 |
| 31 | *Kininogen-1* | 2.61 | *Deoxynucleoside triphosphate triphosphohydrolase SAMHD1* | 2.45 |
| 32 | Proteasome activator complex subunit 1 | 2.52 | Proteasome activator complex subunit 1 | 2.43 |
| 33 | Proteasome subunit alpha type-7 | 2.48 | *[Pyruvate dehydrogenase (acetyl-transferring)] kinase isozyme 4, mitochondrial* | 2.42 |
| 34 | *[Pyruvate dehydrogenase (acetyl-transferring)] kinase isozyme 4, mitochondrial* | 2.45 | Proteasome subunit alpha type-7 | 2.40 |
| 35 | Glucose 1-dehydrogenase, 6-phosphogluconolactonase | 2.41 | Long-chain-fatty-acid--CoA ligase 4 | 2.36 |
| 36 | Inorganic pyrophosphatase | 2.39 | **von Willebrand factor A domain-containing protein 5A** | 2.33 |
| 37 | *Signal transducer and activator of transcription 1* | 2.30 | Prelamin-A/C; Lamin-A/C | 2.30 |
| 38 | **Apolipoprotein E** | 2.28 | Basal cell adhesion molecule | 2.29 |
| 39 | **Serotransferrin** | 2.26 | Glucose 1-dehydrogenase, 6-phosphogluconolactonase | 2.27 |
| 40 | **Serum amyloid A-1 protein** | 2.25 | Adenosine kinase | 2.27 |
| 41 | *Protein S100-A9* | 2.25 | *H-2 class I histocompatibility antigen K-W28 alpha and K-Q alpha chain* | 2.21 |
| 42 | **Vitamin D-binding protein** | 2.24 | *Signal transducer and activator of transcription 1* | 2.21 |
| 43 | Actin-related protein 2/3 complex subunit 2 | 2.23 | *Microsomal glutathione S-transferase 1* | 2.12 |
| 44 | **Serum albumin** | 2.22 | *Macrophage-capping protein* | 2.00 |
| 45 | *High mobility group protein B2* | 2.16 |  |  |
| 46 | **Apolipoprotein A-I** | 2.14 |  |  |
| 47 | ADP-ribosylation factor 4 | 2.08 |  |  |
